# Long-term effect of motor cortex stimulation in patients suffering from chronic neuropathic pain: An observational study

**DOI:** 10.1371/journal.pone.0191774

**Published:** 2018-01-30

**Authors:** Dylan J. H. A. Henssen, Erkan Kurt, Anne-Marie van Cappellen van Walsum, Inge Arnts, Jonne Doorduin, Tamas Kozicz, Robert van Dongen, Ronald H. M. A. Bartels

**Affiliations:** 1 Department of Anatomy, Donders Institute for Brain, Cognition & Behaviour, Radboud University Medical Center, Nijmegen, the Netherlands; 2 Department of Neurosurgery, Radboud University Medical Center, Nijmegen, the Netherlands; 3 Unit of Functional Neurosurgery, Radboud University Medical Center, Nijmegen, the Netherlands; 4 Department of Anesthesiology, Pain and Palliative Care, Radboud University Medical Center, Nijmegen, the Netherlands; 5 Department of Neurology, Radboud University Medical Center, Nijmegen, the Netherlands; Universidade de Sao Paulo Faculdade de Medicina, BRAZIL

## Abstract

**Background:**

Motor cortex stimulation (MCS) was introduced as a last-resort treatment for chronic neuropathic pain. Over the years, MCS has been used for the treatment of various pain syndromes but long-term follow-up is unknown.

**Methods:**

This paper reports the results of MCS from 2005 until 2012 with a 3-year follow-up. Patients who suffered from chronic neuropathic pain treated with MCS were studied. The analgesic effect was determined as successful by decrease in pain-intensity on the visual analog scale (VAS) of at least 40%. The modifications in drug regimens were monitored with use of the medication quantification scale (MQS). Stimulation parameters and complications were also noted. Interference of pain with quality of life (QoL), the Quality of Life Index (QLI), was determined with use of a specific subset of questions from the MPQ-DLV score.

**Results:**

Eighteen patients were included. Mean pre-operative VAS changed from 89.4 ± 11.2 to 53.1 ± 25.0 after three years of follow-up (*P* < 0.0001). A successful outcome was achieved in seven responders (38.9%). All patients in the responder group suffered from pain caused by a central lesion. With regard to all the patients with central pain lesions (n = 10) and peripheral lesions (n = 8), a significant difference in response to MCS was noticed (*P* = 0.002). MQS scores and QLI-scores diminished during the follow-up period (*P =* 0.210 and *P* = 0.007, respectively).

**Conclusion:**

MCS seems a promising therapeutic option for patients with refractory pain syndromes of central origin.

## Introduction

In the early 1990s, Tsubokawa and colleagues presented that chronic motor cortex stimulation (MCS) was effective in treating thalamic pain syndromes[1]. Over the years, various reports in which this last resort treatment was discussed were published and more indications for MCS were introduced; see for reviews, Lima et al. and Fontaine et al.[2, 3]. The main indications for MCS are [1] central post-stroke pain (including thalamus syndrome), [2] neuropathic orofacial pain of various origins, [3] phantom limb pain, and [4] pain due to peripheral plexus avulsion[3, 4]. It has recently been estimated that over 700 patients were treated with MCS worldwide, using a variety of protocols[5]. Due to this heterogeneity, comparison of the results of MCS remains difficult[5]. Next to the many success cases, other papers that discuss the lack of efficacy of MCS have been published as well[6–8]. Nevertheless, the current literature suggests that MCS holds promise for patients that suffer from specific conditions, such as trigeminal neuropathic pain and post-stroke pain[9], although the exact mechanisms of MCS remain matter of debate. One of the hypothesized mechanisms includes the activation of various interneural circuits within the primary motor cortex, inducing an antidromic modulation of the thalamacortical fibers[1, 10, 11]. With regard to this, different forms of stimulation have shown to activate different neural circuits. Anodal stimulation seems to preferably activate the corticospinal tract directly, whereas cathodal stimulation seems to stimulate the thalamocortical tracts and the corticospinal tract indirectly. The stimulation of thalamocortical tracts and indirect stimulation of the corticospinal tract was observed to achieve the greatest pain relief[11]. MCS has also been shown to activate different brain regions remote from the site of stimulation. For example, the orbitofrontal cortex, the insula, the cingulate cortex, the putamen, the thalamus and the PAG have all been observed as areas that are influenced by MCS[12, 13]. Next to these aforementioned mechanisms, the neurochemical effects of MCS has been investigated as well. For example, MCS is known to enhance the release of endogenous opioids in pain-related circuits such as the periaquaductal grey and cingulate cortex[14]. Furthermore, the density of opioid receptor binding in the brain predicts the clinical outcome of MCS[15]. It has also been postulated that the activation of both inhibitory (GABAergic) pathways and excitatory (glutametergic) pathways[11, 16, 17] plays an important role in MCS. For instance, an impaired intracortical inhibition, present in neuropathic pain patients, seems to be restored after stimulation of the primary motor cortex. This indicates the involvement of the inhibitory system[17]. Other studies proposed the involvement of the inhibitory system in thalamic modulation after MCS[18–22]. The excitatory pathways are also hypothesized to be involved as the putamen releases dopamine after MCS as a result of activation of the glutametergic corticostriatal projections[23, 24]. The link between the analgesic effect of MCS and the glutamate N-methyl-D-aspartate (NMDA) receptors has been established as well[25, 26], which could explain the long-lasting analgesia that occurs after stimulation of the motor cortex[27]. Other evidence from experimental models of neuropathic pain show that analgesia after electrical stimulation originates from the rostroventral medulla as well as the descending serotoninergic pathways, which shows involvement of the serotonergic system[28]. Next to the discussion concerning suitable candidates and the influenced pathways, the efficacy of MCS seems to depend greatly on the appropriate positioning of the electrode over the cortical area of interest and the applied stimulation parameters. Different shapes of the electrodes and settings of the pulse generator are known to stimulate different neural structures and mechanisms in the brain[11, 29]. This study aims to determine the efficacy of MCS by presenting the results of MCS after a long-term follow-up of 3 years, using an assessment using changes in VAS scores, the daily intake of pain medication and the changes in quality of life (QoL) scores.

## Material and methods

### Study protocol

In 2003, an observational study protocol was set up in the university medical centers of Nijmegen and Groningen, the Netherlands, in order to study the effects of MCS in patients that suffered from chronic neuropathic pain[30]. Patients were included between 2005 and 2013 when they suffered from chronic intractable neuropathic pain and reported high levels of pain (VAS ≥ 5, measured three times daily during four days[31]). The diagnosis chronic neuropathic pain was based primarily on the patient’s history and physical examination. Questionnaires based on the sensory descriptors and the quality of life have been developed to diagnose chronic neuropathic pain. These instruments have been shown to be valid and reliable discriminators of chronic neuropathic pain. In addition, the presence of weakness, allodynia or hyperalgesia all favor a diagnosis of chronic neuropathic pain[32]. Furthermore, radiographic imaging techniques performed less than three years before inclusion for MCS, a neuroanatomical explanation that might contribute to the pain should be seen. Patients were selected by anesthesiologist-pain specialists, neurosurgeons, and clinical psychologists. At intake, patients were asked to fill-in 1)the McGill Pain Questionnaire; 2)the Symptom CheckList 90; 3)the 5-level Euro Quality of Life 5D version; 4) the Sickness Impact Profile 68 and; 5)to register their daily medication intake using a medication journal. Quantitative sensory testing and an extensive physical examination were carried out to assess and quantify sensory- and motor function. Patients with severe, current psychological problems (e.g., depression, high anxiety) or substance-abuse were excluded. Other exclusion criteria were the use of therapeutic anticoagulants, cognitive and/or psychiatric disorders in the medical history, nociceptive pain, an expected life expectancy less than 3 years due to other diseases (e.g., cancer), contra- indications for general anesthesia (e.g., severe cardio-pulmonary diseases), convulsive disorders and the presence of other neuromodulation systems. All patients underwent preoperative somatosensory-evoked potential (SSEP) measurement to determine the integrity of the somatosensory system in order to facilitate intra-operative neurophysiological monitoring. A MRI-scan was used to determine any anatomical contra-indications (brain atrophy, pathological structures) for the operative procedure. All patients presented in this study had a follow up of three years. Final diagnosis of patients was carried out in accordance with internationally renowned guidelines[33, 34].

### Surgical technique

The pre-operative fMRI was fused with the neuronavigation MRI. For this purpose, cortex surface rendering technique was performed using the Stealthviz software (*Medtronic Inc*., *Minneapolis*, *MN*, *USA*) to visualize the cortical areas and determine the central sulcus and the motor cortex, which then was marked on the skin by using neuronavigation. All patients were operated under general anesthesia without muscle relaxation. A small craniotomy (approximately 4 × 4 cm) was carried out over the central sulcus. An electrode was placed perpendicular to the central sulcus in the epidural space (*Specify*, *model 3998*, *Medtronic Inc*., *Minneapolis*, *MN*, *USA*). The central sulcus was identified using the phase reversal of the somatosensory evoked potential recorded with an eight-contact electrode strip during median nerve stimulation. Consequently, monopolar anodal train-of-five stimulation was used to map motor function at different locations in the precentral gyrus. Stimulation intensity was increased until a reproducible motor evoked potential was found in the muscle of interest (e.g. m. flexor carpi radialis or m. abductor pollicis brevis). Results from electrophysiological testing were taken together with intraoperative neuronavigation to determine the optimal cortical target for MCS. The MCS electrode was sutured to the dura mater. After placement of the electrode, the electrode was tunneled subcutaneously and connected with an internalized pulse generator (IPG) (*Medtronic Versitrel* and later *Prime Advanced*) that was implanted in the subclavian space or in a subcutaneous abdominal pocket.

### Data-analysis

An independent researcher (D.H.), who was blinded to the stimulation conditions, investigated the patient records in this observational study. Only patients who were treated in accordance to the aforementioned treatment protocol and with a minimal follow-up of three years in whom the effect of MCS, occurrence of complications, daily intake of medication and change in quality of life was complete, were analyzed.

### Ethical statement registration of clinical trial and reporting

This observational study was performed under the approval of the medical ethical committee of the region Arnhem–Nijmegen. All patients, after extensive pre-operative information, gave written informed consent due to the experimental nature of this treatment at that time. This clinical trial was not registered in 2003 due to the fact that, in The Netherlands, MCS was not an experimental method at that moment. The authors confirm that all ongoing and related trials for this intervention are registered (ClinicalTrials.gov Identifier: NCT03189823). The TREND Statement Checklist was added to this paper in order to contribute to the standardization of the reporting of non-randomized trials (**S1 File**).

### Assessment

Pain is a complex, subjective and multidimensional phenomenon that is difficult to measure by unidimensional pain scores only. Apart from the visual analog scale (VAS), the intake of pain medication is thought to be a valid tool of measuring pain relief[35, 36]. Adding analgesic drug intake as an outcome parameter could provide a more realistic assessment of long-term benefits of MCS[37]. Five outcome variables were examined: (1) the amount of pain relief, measured by the mean difference between VAS score pre-operatively and the VAS score during the follow-up (1 month, 6 months, 1 year, and 3 years after implantation of the MCS electrodes); (2) the change in the drug regimen of all patients per day; (3) interference of pain with quality of life (QoL); (4) adverse events (infection, bleeding, hardware removal, temporary seizures, and battery dysfunction); and (5) the correlation between stimulation parameters and the pain relief per patient. Pain relief was divided into three categories[38]. A good pain relief, level 1, was defined as a VAS reduction of 70–100%. Reduction of pain according to a VAS scores change between 40% and 69% was defined as satisfactory (level 2), while a minimal pain relief was defined as a reduction of ≤ 40% on the VAS scores. A clinical relevant pain relief was defined as ≥ 40% reduction of pain (levels 1 and 2)[38, 39]. The use of medication was monitored using the electronic patient record during follow-up. The medication quantification scale (MQS) was used in order to quantify medication use and was calculated for each drug by multiplying the dosage levels by their respective detriment weight[40]. The dosage levels (0–6) were based on the recommended daily dosage range as described by Masters Steedman et al.[41]. These scores are summed to provide a quantitative index of total drug intake suitable for statistical analysis. Interference of pain with quality of life (QoL) was measured before and after (> 1 year) MCS with use of the Quality of Life Index (QLI), based on the Dutch version of the McGill pain questionnaire (MPQ-DLV)[42, 43]. The occurrence of complications was documented as well. Apart from biological complications (eg. bleeding, infection), the removal of the hardware due to a minimal effect was evaluated as well. To determine whether there was a correlation between the used stimulation parameters and the pain relief, the used stimulation parameters (intensities [V], pulse widths [μs], and frequencies [Hz]) were reviewed.

### Statistical analysis

IBM SPSS Statistics version 22 was used for statistical analyses of the retrieved data (*IBM Corp*. *Released 2013*. *IBM SPSS Statistics for Windows*, *Version 22*.*0*. *Armonk*, *NY*: *IBM Corp*.). The differences in VAS-scores MQS-scores and QoL-indices over time were analyzed using a mixed model analysis. To determine differences between groups in MQS-scores, the Mann-Whitney *U* test was used. In order to correlate the applied stimulation parameters, the Spearman rank correlation coefficient was conducted. To determine the complication rate across time, a Poisson regression analysis was conducted. Values are represented as mean ± standard deviation (minimum- maximum). Statistical tests were two sided and with a significance level of *P* < 0.05 (**S2 and S3 Files**).

## Results

**Fig 1:**
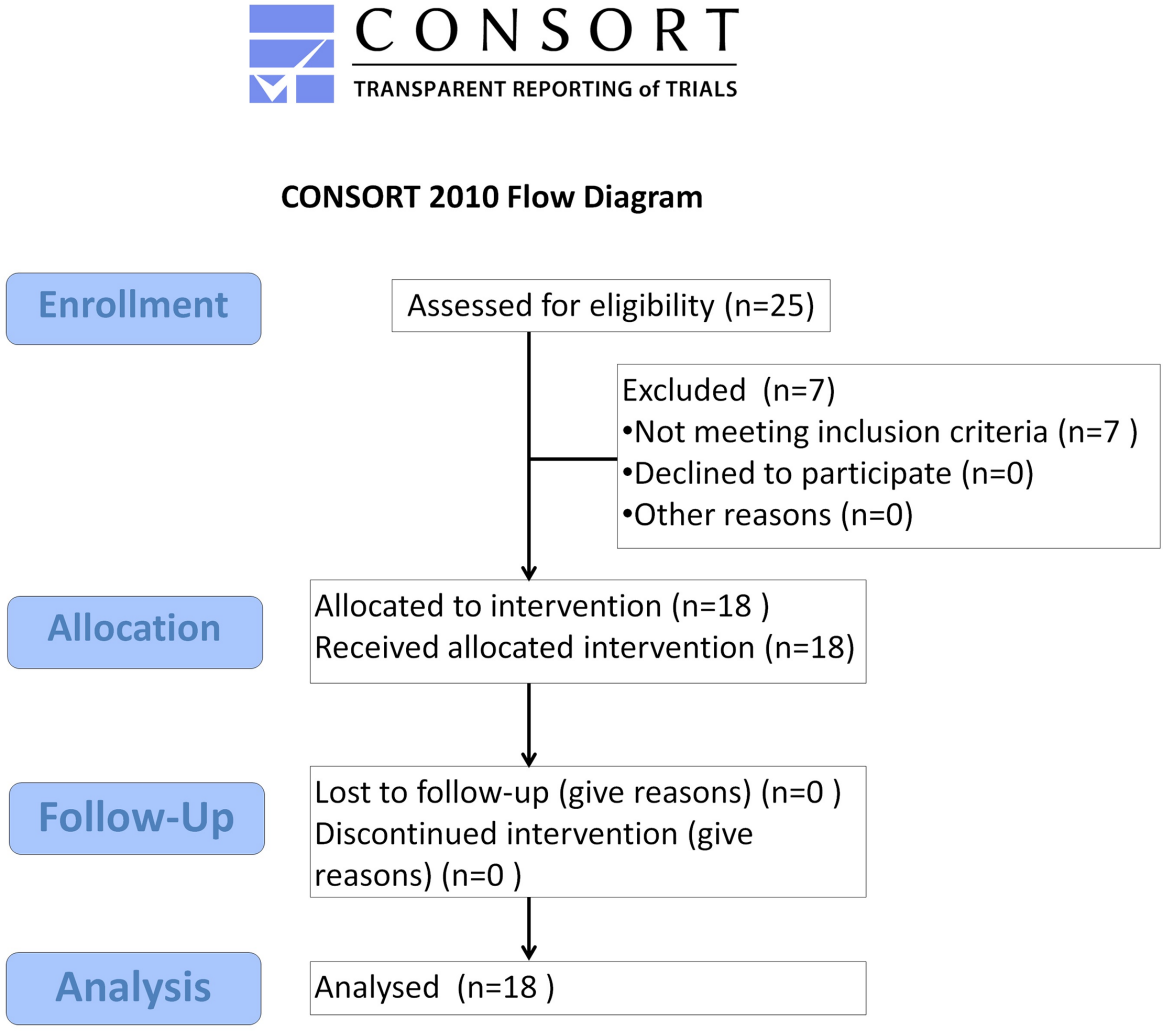
CONSORT 2010 flow diagram.

Eighteen patients were included (**Fig 1**). The mean age was 59.0 ± 7.3 years (41–72 years), and 10 of them were females. The mean duration of pain was 7.7 ± 6.1 years (2–26 years). **Tables 1 and 2** summarize the baseline surgery-related characteristics and the outcomes of MCS and the complications due to surgery. The complete, anonymized database has been made available; see **S1 Database**).

**Table 1:** Demographic characteristics.

	Sex/ age, years	Cause	Diagnosis	Group^a^	Location of pain	Duration of pain, years	Sensory loss	Allodynia	Motor weakness	Timing	Pain descriptors
1	M/58	WS	PSP	A	Right hemiface and left hemibody	3	Yes	Yes	No	Continuous	Aching; Pricking
2	M/65	CVA	PSP	A	Left hemibody	4	Yes	Yes	No	Continuous	Burning
3	M/61	WS	PSP	A	Left hemiface	16	Yes	Yes	Yes	Continuous	Burning
4	F/67	CVA	PSP	A	Left hemiface and tongue	26	Yes	Yes	Yes	Continuous	Aching
5	M/61	TN	TN1	A	Left hemiface	13	Yes	No	No	Paroxysmal	Electric; Sharp; Shooting
6	M/56	TN	TN1	A	Left hemiface	12	Yes	Yes	Yes	Paroxysmal	Aching; Electric; Sharp
7	M/57	TN	TN2	A	Left hemiface	10	Yes	Yes	No	Continuous	Aching; Electric; Sharp
8	F/50	TS	PSP	B	Left hemibody	6	Yes	Yes	No	Continuous	Dull; Throbbing
9	M/59	CVA	PSP	B	Left hemiface and left hand	8	Yes	Yes	Yes	Continous	Burning
10	F/52	CVA	PSP	B	Right hemiface and right arm	2	Yes	Yes	Yes	Continous	Burning
11	F/41	X	PIFP	B	Left hemiface	9	No	Yes	No	Continuous	Aching; Dull; Nagging
12	F/63	X	PIFP	B	Left hemiface	4	No	Yes	No	Continuous	Dull; Nagging
13	F/63	ESG	CPSP	B	Left hemiface and left tongue	5	Yes	Yes	No	Paroxysmal	Electric; Shooting
14	F/72	IONP	BMS	B	Right tongue and oral cavity	6	No	Yes	No	Continuous	Burning
15	F/54	MF	TNP	B	Left hemiface	6	Yes	Yes	No	Continuous	Burning; Electrical
16	F/61	IONP	BMS	B	Intraoral mucosa	2	No	No	No	Continuous	Burning
17	F/54	AMP	PhP	B	Phantom limb pain of right arm	3	N/A	N/A	N/A	Continuous	Burning; Cramping
18	M/68	BPA	AvP	B	Left arm	3	Yes	No	Yes	Continuous	Burning; Shooting

^a^ Group A, responding patients; group B, non-responding patients

AMP, amputation; AvP, avulsion pain; BMS, burning mouth syndrome; BPA, brachial plexus avulsion; CPSP: Chronic post-surgical pain; CVA, cerebrovascular accident; ESG, extirpation of the submandibular gland; F, female; IONP, intraoral neuropathic pain; M, male; MCS, motor cortex stimulation; MF, mandibular fracture; PSP, post-stroke pain; PIFP, persistent idiopathic facial pain; TN1, trigeminal neuralgia type 1 (>50% episodic); TN1, trigeminal neuralgia type 1 (>50% constant); TNP, trigeminal neuropathic pain; TS, thalamus syndrome; VAS, visual analog scale; WS, Wallenberg syndrome; X, unknown

**Table 2:** VAS scores and complication registration.

	Sex/ age, years	Group^a^	VAS before MCS	VAS 1 month	Pain relief%	VAS 6 months	Pain relief%	VAS 1 year	Pain relief%	VAS 3 years	Pain relief%	Level of pain control^b^	Device removed	Complications
1	M/58	A	50–80	30	54	30	54	30	54	30	54	2	Yes	Infection
2	M/65	A	90	20–30	72	30	67	40	56	40	56	2	Yes	Infection
3	M/61	A	100	60–70	35	60–70	35	40–70	45	40	60	2	No	
4	F/67	A	100	20	80	20	80	40	60	30	70	1	No	
5	M/61	A	80	30	63	30	63	0	100	0	100	1	No	
6	M/56	A	90	40	56	40	56	30	67	30	67	2	No	
7	M/57	A	100	60	40	50	50	50	50	40	60	2	No	Temporary seizures
8	F/50	B	60–90	60	20	60	20	50	33	60	20	3	Yes^c^	
9	M/59	B	80	60	25	50	38	60	25	50	38	3	No	
10	F/52	B	100	80–100	10	80	20	60	40	80	20	3	Yes^c^	
11	F/41	B	100	80	20	70	30	60–80	30	80	20	3	Yes^c^	
12	F/63	B	80	40–80	25	40–80	25	40–80	25	40–80	25	3	No	
13	F/63	B	100	10–20	85	10–20	85	70–80	25	70–80	25	3	No	IPG hardware malfunctioning
14	F/72	B	80	60–80	13	80	0	70	12.5	80	0	3	No	
15	F/54	B	100	80	20	80	20	80	20	80–100	10	3	No	
16	F/61	B	90	70	22	80	11	80	11	80	11	3	Yes^c^	
17	F/54	B	80	70	12.5	70	12.5	70–80	7.5	70	12.5	3	Yes	Infection
18	M/68	B	100	70	30	70	30	70	30	70	30	3	No	

^a^ Group A, responding patients; group B, non-responding patients

^b^ Level 1, 70–100%; level 2, 40–69%; level 3, 0–39%.

^c^ Device removed due of minimal effect according to patients perspective.

F, female; IPG, implantable pulse generator; M, male

### Pain relief according to VAS scores

The mean pre-operative VAS was 89.4 ± 11.2. A mean VAS score of 59.2 ± 21.4 was observed after 1 month (*P* < 0.0001). A clinical relevant pain relief (level 1 and 2) was observed in 38.9% of the patients after 1 month. After 6 months, a mean VAS score of 58.3± 20.9 was observed (*P* < 0.0001). After 1 year, the most optimal results were observed with a clinical relevant pain relief in 44.4% of the patients and a mean VAS score of 56.1 ± 29.6 (*P* < 0.0001). After 3 years of MCS, a mean VAS score of 53.1 ± 25.0 was observed (*P* < 0.0001).

A clinical relevant pain relief was observed in 38.9% of the described population after 3 years of follow-up and a mean VAS reduction of 36.4 points. This indicates seven responders (R) to MCS and eleven non-responding patients (NR). All responders to MCS (n = 7) showed to be suffering pain due to lesions in the central nervous system. In the non-responder group (n = 11), central lesions were seen in three cases. With regard to all the patients with central pain lesions (n = 10), a clinical relevant pain relief was observed in 70% of the cases and a mean VAS reduction of 54.5% ± 23.9 could be observed. All the patients with a peripheral lesion (n = 8) showed a mean VAS reduction of 16.7% ± 10.0. This indicated a significant difference in response to MCS (*P* = 0.002). As many of the patients suffered from orofacial pain, this group was also reviewed separately. When the orofacial pain candidates and the site of the lesion were reviewed, MCS showed to have a significant favorable outcome (*P* = 0.003) in the treatment of proximal (or central) lesions (**Table 3**).

**Table 3:** Characteristics of the orofacial pain patient group.

	Sex/ age, years	Painful area	L/R	Diagnosis	Group^a^	Period of pain, years	Sensory loss	Allodynia	Motor deficit	History of interventions/problems with regard to the orofacial pain syndrome	Pain relief, (%, after 3 years of MCS	Level of pain control^b^	Changes in MQS^c^	Changes in QLI^d^
**1**	M/58	Hemiface and hemibody	R+L*	PSP	A	3	Yes	Yes	No	Rhinosinal surgery; sweet procedure	54	2	Diminished	N/A
**3**	M/61	Hemiface	R+L*	PSP	A	16	Yes	No	No	Sweet procedure; thermolesion; ITB therapy	60	2	Diminished	Diminished
**4**	F/67	Hemiface	L	PSP	A	26	Yes	Yes	Yes	TENS; multiple PEA injections; Dandy procedure	70	1	Diminished	Diminished
**5**	M/61	Hemiface	L	TN1	A	13	Yes	Yes	Yes	TENS; PEA injection; Sweet procedure; 2x vascular decompression; stereotactic radiosurgery	100	1	Diminished	Diminished
**6**	M/56	Hemiface	L	TN1	A	12	Yes	Yes	Yes	3x stereotactic radiosurgery	67	2	No change	Diminished
**7**	M/57	Hemiface	L	TN2	A	10	Yes	Yes	No	Glycerol injection; vascular decompression; stereotactic radiosurgery	60	2	Diminished	Diminished
**9**	M/59	Hemiface and hemibody	L	PSP	B	8	Yes	Yes	Yes	N/A	38	3	Diminished	Diminished
**10**	F/52	Hemiface and hemibody	R	PSP	B	2	Yes	Yes	Yes	N/A	20	3	Increased	No change
**11**	F/41	Hemiface	L	PIFP	B	9	No	Yes	No	Vascular decompression; rhinosinal surgery; mandibular surgery; 5x Sweet procedure	20	3	Diminished	Diminished
**12**	F/63	Hemiface	L	PIFP	B	4	Yes	Yes	No	Vascular decompression; sinus surgery	25	3	Increased	No change
**13**	F/63	Hemiface	L	CPSP	B	5	Yes	Yes	Yes	Submandibular surgery; PEMF; 3x RF lesion	25	3	Increased	Diminished
**14**	F/72	Intraoral	R	BMS	B	6	No	Yes	No	Malfitting dentures; vascular decompression of n. V, n. IX and n. X	0	3	Increased	Diminished
**15**	F/54	Hemiface	L	TNP	B	6	Yes	Yes	No	Vascular decompression; stereotactic radiosurgery,	10	3	Diminished	Diminished
**16**	F/61	Intraoral	R+L	BMS	B	2	No	No	No	18x dental surgery	11	3	No change	No change

^a^ Group A, responding patients; group B, non-responding patients

^b^ Level 1, 70–100%; level 2, 40–69%; level 3, 0–39%.

^c^ Dimished, MQS after MCS<MQS before MCS (negative rank); Increased, MQS after MCS>MQS before MCS (positive rank); No change, MQS after MCS equal to MQS before MCS (tie).

^d^ Dimished, QLI after MCS<QLI before MCS (negative rank); Increased, QLI after MCS>QLI before MCS (positive rank); No change, QLI after MCS equal to QLI before MCS (tie).

* In Wallenberg syndrome or infarction of brainstem and cerebellum often causes bilateral pain; (oro)facial pain will occur ipilateral to the side of the lesion; pain in the body will occur contralateral to the side of the lesion

CPSP: Chronic post-surgical pain; F, female; ITB, intrathecal baclofen therapy; L/R, left or right; M, male; MCS, motor cortex stimulation; MQS, medication quantification scale; N/A, not applicable/not available; PEA, palmitoylethanolamide injection; PEMF, pulsed electromagnetic field stimulation; PSP, post-stroke pain; PIFP, persistent idiopathic facial pain; RF lesion, radiofrequent nerve lesion; TENS, transcutaneous electroneurostimulation; TN1, trigeminal neuralgia type 1 (>50% episodic); TN1, trigeminal neuralgia type 1 (>50% constant); TNP, trigeminal neuropathic pain; AvP, avulsion pain; BMS, burning mouth syndrome

### Modifications in drug regimens

**Fig 2:**
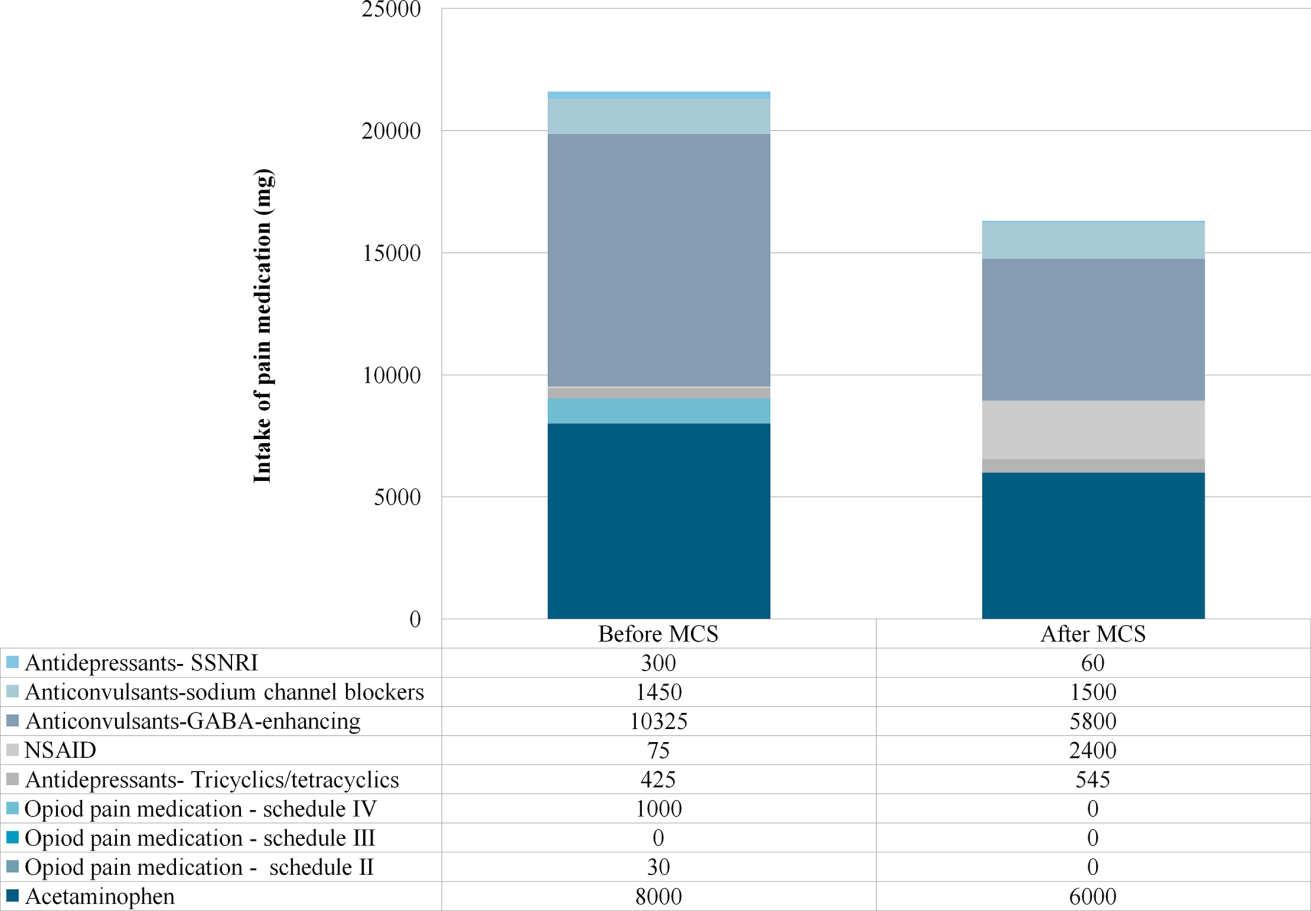
Differences in the cumulative, total intake of pain medication in milligram per day before and after MCS. GABA, gamma-aminobutyric acid; NSAID, non-steroidal anti-inflammatory drug; MCS, motor cortex stimulation; SSNRI, selective serotonin and norepinephrine reuptake inhibitor.

At baseline, 16 patients used pain medication. **Fig 2** discloses the cumulative daily intake of pain medication before and after MCS. A total reduction of 24% is shown. The daily intake of opioids decreased to zero after MCS. MQS scores per patient before and after MCS are represented in **Table 4**. The median MQS score before MCS was 6.6, whereas the median MQS score after MCS was 5.4, which did not reach statistical significance (*P =* 0.210). Responders showed a median MQS score before and after MCS of 6.3 and 5.4, respectively, ranging from 0 to 14.1 before MCS and 0 to 20.7 after MCS (z = -0.734; *P =* 0.463). The non-responders had a median MQS score of 11.5 and 5.4 before and after MCS, respectively, ranging from 5.4 to 26.1 before and 0 to 15.6 after MCS (z = -2.549; *P =* 0.011).

**Table 4:** Stimulation parameters, medication quantification scales and analgesic effects per patient.

	Diagnosis	Group^a^	Pain relief, %, after 3 years of MCS	Level of pain control^b^	Intensity, V^c^	Frequency, Hz^c^	Pulse width, μs^c^	MQSpre	MQSpost	Changes in MQS^d^	QLI pre	QLI post	Changes in QLI^e^
1	PSP	A	54	2	2	50	90	16.5	4.2	Diminished	N/A	N/A	N/A
2	PSP	A	56	2	1.5	50	80	5.4	5.4	No change	N/A	N/A	N/A
3	PSP	A	60	2	2.5	50	60	15.3	13.2	Diminished	9	7	Diminished
4	PSP	A	70	1	2.5	60	60	6.3	4.2	Diminished	14	2	Diminished
5	TN1	A	100	1	3	50	60	8.2	0	Diminished	7	2	Diminished
6	TN1	A	67	2	4.5	40	90	0	0	No change	14	4	Diminished
7	TN2	A	60	2	4.5	50	120	6.3	4.8	Diminished	8	4	Diminished
8	PSP	B	20	3	2.5	50	90	26.1	4.2	Diminished	7	1	Diminished
9	PSP	B	38	3	3	50	60	14.1	4.2	Diminished	11	10	Diminished
10	PSP	B	20	3	2.5	50	60	0	5.4	Increased	16	16	No change
11	PIFP	B	20	3	4	50	60	11.5	8.4	Diminished	16	12	Diminished
12	PIFP	B	25	3	5	50	90	6.3	6.9	Increased	13	13	No change
13	CPSP	B	25	3	3	50	60	6.4	7.8	Increased	16	8	Diminished
14	TNP	B	0	3	3	40	60	6.8	10.7	Increased	12	10	Diminished
15	TNP	B	10	3	2	40	80	23.4	15.6	Diminished	11	10	Diminished
16	BMS	B	11	3	3	40	90	5.	5.4	No change	8	8	No change
17	PhP	B	12.5	3	2.5	50	60	6.3	12	Increased	N/A	N/A	N/A
18	AvP	B	30	3	3	50	60	24.6	14.2	Diminished	17	8	Diminished

^a^ Group A, responding patients; group B, non-responding patients

^b^ Level 1, 70–100%; level 2, 40–69%; level 3, 0–39%.

^c^ Stimulation parameters that were reported at the last follow-up.

^d^ Dimished, MQS after MCS<MQS before MCS (negative rank); Increased, MQS after MCS>MQS before MCS (positive rank); No change, MQS after MCS equal to MQS before MCS (tie).

^e^ Dimished, QLI after MCS<QLI before MCS (negative rank); Increased, QLI after MCS>QLI before MCS (positive rank); No change, QLI after MCS equal to QLI before MCS (tie).

AvP, avulsion pain; BMS, burning mouth syndrome; BPAP, brachial plexus avulsion pain; CPSP: Chronic post surgical pain; Hz, Hertz; μs, microseconds; MCS, motor cortex stimulation; MQS, medication quantification scale; MQSpost, medication quantification scale following MCS; MQSpre, medication quantification scale before MCS; N/A, Not available; PIFP, persistent idiopathic facial pain; PSP, post-stroke pain; QoL-index pre, Quality of life index before MCS; QoL-index post, Quality of life index following MCS; TN1, trigeminal neuralgia type 1 (>50% episodic); TN1, trigeminal neuralgia type 1 (>50% constant); TNP, trigeminal neuropathic pain

### Quality of life index

The QoL-index was used in order to measure the impact of MCS on the pain-related quality of life. QoL-indices were retained from thirteen patients. Three patients were lost during follow-up of QoL due to recurrent infections and removal and re-implantation of MCS electrodes (#1;2;17). The mean pre- and postoperative QoL-index showed to be 11.9 ± 3.5 and 7.7 ± 4.4, indicating a significant improvement in QoL (*P* = 0.007). Responders and non-responders to MCS did not show a significant different preoperative QoL-index (*P* = 0.249), whereas the postoperative QoL-index showed significant differences in favor of the group responders (*P* = 0.002).

### Complications

Complications occurred in nine patients (50%). In three patients, an infection of the electrode and the extension wire occurred and the electrodes were removed (#1;2;17). One patient (#10) experienced a short-lasting, intra-operative epileptic seizure during intra-operative stimulation of the motor cortex. One patient suffered from an IPG hardware malfunctioning (#13). In four cases, the system was explanted on request of the patient due to an unsatisfactory pain relief (#8;10;11;16). After it was observed that some patients suffered from local pain after implanting the IPG in the subclavian area, the IPG implantation area was switched to the subcutaneous abdominal space (#3;5;7). The complication rate across time showed to be 0.16 events per person years (*95%-Confidence interval =* 0.07–0.34).

### Stimulation parameters

The intensity of the stimulation varied between 1.5 V and 5.0 V. The frequencies ranged from 40 Hz to 60 Hz, and the pulse width ranged from 60 μs to 120 μs (**Table 4**). No correlation between the applied intensities or pulse widths and the pain relief at last follow-up could be observed (Spearman correlation = 0.1, *P* = 0.692, and Spearman correlation = 0.045, *P* = 0.860, respectively). A significant correlation (*P* = 0.035), however, was observed between the applied frequencies and the pain relief at the last follow-up (*Spearman correlation* = 0.498).

## Discussion

### Effect of MCS

This paper reports that the positive effects of MCS are associated with a decrease in pain scores (VAS), improved quality of life and a diminished consumption of pain medication. Various reports in chronic pain management show that the improvement that patients experience after MCS takes place in numerous domains. Therefore, the analgesic effects must be registered using pain intensity scores, quality of life assessments and a medication intake score [44]. For example, this study shows that, although some patients do not report a significant pain reduction, their pain medication diminished. This weaning of opioids can be the effect of MCS, but it can also be causative as during follow-up, patients were screened for the effects and side-effects of the analgesics they used. Some patients could gradually be weaned from opioids due to inefficacy and bothersome side-effects. Therefore, as stated earlier, a single change in outcome in these complex pain syndromes simplifies the quality of the treatment effect. The QLI showed to be significantly decreased by MCS in all patients, which is an aspect that is not frequently addressed in the literature on MCS.

### Central vs. peripheral pain

As mentioned, a significant difference in response to MCS between the central and peripheral pain patients was observed. This phenomenon could be the result of a hypothesized double projection of body regions to the thalamus and the somatosensory cortical areas[45, 46]. If both thalami get involved into nociceptic processing due to a peripheral lesion, MCS might not be capable to provide a complete analgesic effect in this group. The significant difference in response to MCS in orofacial pain could be the result of a recent review, hypothesizing a double tract that conducts orofacial pain[47]. However, in general, classic trigeminal neuralgia is not considered an etiology which is treatable by MCS. Due to the extensive history of interventions to provide pain relief, including glycerol- and palmitoylethanolamide injections, stereotactic radiosurgery, Sweet procedures and vascular decompressions, the diagnosis of a classic trigeminal neuralgia can be discussed. All the aforementioned interventions possible damage the trigeminal nerve, which would lead trigeminal neuropathic pain, instead of pain caused by a classic trigeminal neuralgia. In short, the onset of the trigeminal neuralgias in all these patients was most probably idiopathic but the chronic orofacial pain treated by MCS probably resulted from sensory deafferentation due to various (**Table 3**). Possibly, the combination of a clear anatomical region with detectable changes on neuro-imaging, combined with a relatively large somatotopic area to be stimulated, is key to these findings. However, classifying this anatomical diagnosis in a mechanism-based way remains difficult.

### Stimulation parameters and clinical effects

The various stimulation parameters (intensity, pulse width, frequency) in combination with the anatomical position (e.g. distance dura-cerebral cortex) of the electrode and per-operative neurophysiological measurements, all influence the effects of MCS[11]. Regarding the stimulation parameters used, recent research shows a wide range of parameters[48]. By changing these parameters in each individual case during follow-up and using the (subjective) patient’s response, it is considered that the optimal effect can be reached. Next to the electrical parameters, the type of electrode and lead configuration are thought to be of great importance in the achieved pain reduction[1, 29, 49–51]. No consensus on these matters has been achieved although stimulation over the anterior bank of the central sulcus is frequently reported to provide the best analgesic effect, which can also temporarily be observed in some patients with non-invasive, transcranial magnetic motor cortex stimulation[52]. The use of fMRI and intra-operative cortical mapping as presented in this study seems to be a useful method of finding the optimal target for MCS[53].

### Strengths and limitations

All patients were seen by a team of anesthesiologist-pain specialists, neurosurgeons, and clinical psychologists preceding the operation and during the treatment process. Since it is known that an evaluation by the operator involved creates bias, the participation of an independent observer adds to the strength of the results. The absence of a control group forms an important limitation of this study. Nevertheless, this lack of a control group can be explained by the unacceptable safety aspects of performing a sham surgical intervention and limited trustworthiness of a control group in MCS studies. A double blinded on/off-phase trial could be a valuable addition with regard to the lack of a control group. Second, as over the years new insights in neurophysiological features of chronic pain and MCS were gained, important diagnostic steps were not included in this protocol. For example, Rasche and Tronnier suggested that a double-blinded test trial with an external stimulation device could identify non-responders and placebo responders, hence improving the results of MCS[5]. They also perform a trial with an externalized epidural lead, although the authors think that the effects of MCS can take for months to occur, which makes an externalized epidural lead hazardous due to high risk of infections[5]. Other studies suggest that the efficacy of MCS relies on the number of available opioid receptors in the brain[14, 15]. As both the double-blinded test trial with transcranial stimulation and the pre-operative (11)C-diprenorphine positron emission tomography scan were not widely accessible exploration possibilities, possible non-responders were not recognized. However, these sophisticated techniques are still not widely available.

Nowadays, all ongoing and related trials for MCS must be registered, but just like this study, some studies might not have been registered as this was not common practice at that time. For studies that were started at the time when the registration of trials started and of which the results currently can be presented, this might offer a problem. Strict adherence to this policy might introduce loss of a huge amount of valuable data that could be used to raise questions and help to develop or refute treatments.

Furthermore, more sophisticated imaging techniques such as diffusion tensor imaging, at higher resolutions, could contribute to our understanding of nociceptive pathways in humans. For example, as this report shows, different forms of orofacial pain, conducted by different nociceptive pathways (i.e. central vs. peripheral orofacial pain) respond differently to MCS. A stricter guideline or protocol of selecting and treating patients with MCS seems to be of great importance in order to optimize the efficacy.

## Conclusion

MCS shows to be promising with regard to the long-term effects in patients suffering from chronic, intractable, neuropathic pain, especially in patients who suffer from pain, caused by a central lesion. Optimizing the pre-operative diagnostic procedure and careful patient selection can increase the success rate of MCS. Second, the effect of MCS should not only be evaluated by measuring pain scores alone, but also by alterations in daily intake of pain medication and quality of life.

## Supporting information

S1 File TREND statement checklist. The Transparent Reporting of Evaluations with Non-randomized Designs (TREND) group aimed to improve the reporting standards of non-randomized evaluations of behavioral and public health interventions. The TREND statement checklist is a 22-item checklist that was specifically developed to guide standardized reporting of non-randomized trials. The TREND statement checklist complements the widely adopted CONsolidated Standards Of Reporting Trials (CONSORT) statement, which was developed for randomized controlled trials.
(PDF)

S2 File Clinical protocol in English. (PDF)

S3 File Clinical protocol in Dutch. (PDF)

S1 Database Anonymized database. (XLSX)
