# Supplementary material for: Long-term effect of motor cortex stimulation in patients suffering from chronic neuropathic pain: An observational study
Source: PLoS One. 2018 Jan 30;13(1):e0191774. doi: 10.1371/journal.pone.0191774 (PMC5790239; doi:10.1371/journal.pone.0191774)
Supplement: S2 File — (PDF) [file pone.0191774.s002.pdf]

A PROSPECTIVE STUDY OF THE SAFETY AND EFFICACY OF MOTOR CORTEX STIMULATION (MCS) WITH REGARD TO TREATING PAIN

RADBOUD UNIVERSITY MEDICAL CENTER; NIJMEGEN; THE NETHERLANDS

DONDERS INSTITUTE FOR BRAIN, COGNITION AND BEHAVIOUR; NIJMEGEN; THE NETHERLANDS

UNIVERSITY MEDICAL CENTER GRONINGEN; GRONINGEN; THE NETHERLANDS

UMC ST RADBOUD

Introduction
In 2003, the university medical centers of Nijmegen and Groningen start an observational study in the Netherlands in order to study the effects of MCS in patients that suffered from chronic neuropathic pain.

Indications
1) Central neuropathic pain
2) Orofacial pain

Aim
1) To determine the efficacy of MCS on pain intensity in a clinical setting on short-term, middle-term and long-term follow up (up until three years)
2) To determine the effects of MCS on medications in daily intake of pain medication
3) To determine the effects of MCS on the quality of life before and after treatment
4) To register the safety of MCS with regard to the occurrence of complications

Study design
The study design concerns an open, observational study.

Inclusion criteria
Patients are included when they:
1) suffer from chronic intractable pain
2) report high levels of pain (VAS > 5, measured three times daily during four days)
3) have had radiographic imaging techniques performed less than three years before inclusion for the implantation of MCS to show a possible conflict that might contribute to the pain.
4) are prepared to actively engage in this study and the follow-up

A multidisciplinary approach by the anesthesiologist-pain specialists, neurosurgeons, and clinical psychologists was chosen for the selection of patients.

Exclusion criteria
1) therapeutic anticoagulants
2) severe, current psychological problems (e.g., depression, high anxiety)
3) substance-abuse
4) cognitive and/or psychiatric disorders in the medical history
5) nociceptive pain
6) an expected life expectancy less than 3 years due to other diseases (e.g., cancer)
7) contra- indications for general anesthesia (e.g., severe cardio-pulmonal diseases)
8) convulsive disorders
9) the presence of other neuromodulation systems
10) severe cerebral atrophy

Procedure

All patients undergo an intake session at the outpatient clinic. Furthermore, preoperative somatosensory-evoked potential (SSEP) measurement will be used to determine the integrity of the somatosensory system in order to facilitate intraoperative neurophysiological monitoring. A preoperative MRI- and fMRI- scan is used to determine any anatomical contra-indications (brain atrophy, pathological structures) for the operative procedure. The pre-operative fMRI was fused with the neuronavigation MRI. For this purpose, cortex surface rendering technique will be performed using the Stealthviz software (Medtronic Inc., Minneapolis, MN, USA) to visualize the cortical areas and determine the central sulcus and the motor cortex, which then is marked on the skin by using neuronavigation. All patients will be operated under general anesthesia without muscle relaxation. A small craniotomy (approximately 4 × 4 cm) will be carried out over the central sulcus. An electrode will be placed perpendicular to the central sulcus in the epidural space (Specify, model 3998, Medtronic Inc., Minneapolis, MN, USA). A phase-reversal somatosensory-evoked potential is used intraoperatively to confirm the position of the central sulcus, thereafter cortical stimulation will be performed to map functional motor areas. Following identification of the optimal target, the electrode will be sutured to the dura mater. After placement of the electrode, the electrode is tunneled subcutaneously and connected with an internalized pulse generator (IPG) (Medtronic Versitrel and later Prime Advanced) that will be implanted in the subclavian space or in a subcutaneous abdominal pocket.

Data-analysis

An independent researcher, who will be blinded to the stimulation conditions, investigates the patient records in this observational study.

Ethical statement and registration of clinical trial

This observational study will be performed under the approval of the medical ethical committee of the region Arnhem–Nijmegen. All patients, after extensive pre-operative information, will be asked for written informed consent due to the experimental nature of this treatment at that time. This clinical trial was registered at ClinicalTrials.gov (NCT03189823). The authors confirm that all ongoing and related trials for this intervention are registered

Assessment

Pain is a complex, subjective and multidimensional phenomenon that is difficult to measure by unidimensional pain scores only. Apart from the visual analog scale (VAS), the intake of pain medication is thought to be a valid tool of measuring pain relief. Adding analgesic drug intake as an outcome parameter could provide a more realistic assessment of long-term benefits of MCS. Four outcome variables will be examined: 1) the amount of pain relief, measured by the mean difference between VAS score pre-operatively and the VAS score during the follow-up (1 month, 6 months, 1 year, and 3 years after implantation of the MCS electrodes); 2) the change in the drug regimen of all patients per day; 3) adverse events (infection, bleeding, hardware removal, temporary seizures, and battery dysfunction); and 4) the correlation between stimulation parameters and the pain relief per patient. Pain relief was divided into three categories. A good pain relief, level 1, was defined as a VAS reduction of 70–100%. Reduction of pain according to a VAS scores change between 40% and 69% was defined as satisfactory (level 2), while a minimal pain

relief was defined as a reduction of ≤ 40% on the VAS scores. An effective pain relief is defined as ≥ 40% reduction of pain (levels 1 and 2).

The use of medication will be monitored using the electronic patient record during follow-up. The medication quantification scale (MQS) will be used in order to quantify medication use and will be calculated for each drug by multiplying the dosage levels by their respective detriment weight. The dosage levels (0–6) are based on the recommended daily dosage range as described by Masters Steedman et al. These scores are summed to provide a quantitative index of total drug intake suitable for statistical analysis.

The occurrence of complications will be documented as well. Apart from biological complications (eg. bleeding, infection), the removal of the hardware due to a minimal effect was evaluated as well.

To determine whether there is a correlation between the used stimulation parameters and the pain relief, the used stimulation parameters (intensities [V], pulse widths [µs], and frequencies [Hz]) will be reviewed.

Interference of pain with quality of life (QoL) will be measured before and after (> 1 year) MCS with use of the Quality of Life Index (QLI), based on the Dutch version of the McGill pain questionnaire (MPQ-DLV).

Statistical analysis
IBM SPSS Statistics version 22 is used for statistical analyses of the retrieved data (IBM Corp. Released 2013. IBM SPSS Statistics for Windows, Version 22.0. Armonk, NY: IBM Corp.). To analyze the differences in pain relief and MQS scores and QoL-indices per subgroup, the Mann-Whitney U test will be used. In order to correlate the applied stimulation parameters, the Spearman rank correlation coefficient will be conducted. Values are represented as mean ± standard deviation (minimum- maximum). Alterations in MQS scores and QoL-indices before and after MCS will be calculated with use of the Wilcoxon signed-rank test. Statistical tests are two sided and with a significance level of $P < 0.05$.

Follow-up
Modifications in pain intensity will be measured preoperatively and after 1, 6, 12 and 36 months. During the 36 months of follow-up, the occurrence of complications will be monitored. Modifications in MSQ, QoL-index and correlations between stimulation parameters and pain relief are investigated at 36 months of follow-up.
