# Supplementary material for: Long-term effect of motor cortex stimulation in patients suffering from chronic neuropathic pain: An observational study
Source: PLoS One. 2018 Jan 30;13(1):e0191774. doi: 10.1371/journal.pone.0191774 (PMC5790239; doi:10.1371/journal.pone.0191774)
Supplement: S3 File — (PDF) [file pone.0191774.s003.pdf]

# Protocol:
# Een Pilot study naar de effectiviteit van Motor Cortex Stimulatie (MCS) bij centrale pijnsyndromen

## Versie 6: september 2003

**Instituut voor Anesthesiologie, Pijnbehandelcentrum**
**Afdeling Neurochirurgie**
**Afdeling Neurologie/ Klinische Neurofysiologie**
**F.C. Dondersinstituut**
**UMC St Radboud Nijmegen**
**En**
**Afdeling Neurochirurgie Academisch Ziekenhuis Groningen**

# Effectiviteit van Motor Cortex Stimulatie (MCS) bij centrale pijnsyndromen.

## Inleiding

Pijn na een bloeding of infarct (CVA) in de hersenen of het proximale deel van het ruggenmerg ("centrale pijn") is bijzonder therapieresistent en invaliderend. Ondanks vele interventies, uitgebreide medicamenteuze therapie (alle varianten van pijnstillers, anti-epileptica, antidepressiva etc.) is er vaak een enorme lijdensdruk aanwezig bij familie en patiënt. Min of meer bij toeval werd ontdekt dat elektrische stimulatie van de motorische hersenschors bij proefdieren een pijnstillend effect had bij pijnsyndromen na hersenbeschadiging (1). Er zijn de laatste tiental jaren een aantal klinische studies verricht die het aannemelijk maken dat elektrische stimulatie van de motorische hersenschors (MCS) een pijnmodulerend effect zou kunnen hebben (2-3).
MCS (*motor cortex stimulatie*) is een invasieve techniek waarbij via een ingebrachte (epidurale) intracraniële elektrode en daaropvolgende elektrostimulatie, pijn aan de contralaterale lichaamshelft te beïnvloeden is. De te gebruiken chirurgische techniek verschilt per centrum. Vanwege de incidenteel moeilijk voorspelbare lokalisatie van de elektrode wordt in een aantal centra een relatief grote operatie verricht met mogelijk grotere risico's na de ingreep. Een minimaal invasieve ingreep is te prefereren; echter deze mogelijkheid is afhankelijk van de te voorspellen, exacte lokalisatie van de elektrode.

Historisch gezien zijn er twee indicaties: pijn in een (deel van een) lichaamshelft na een bloeding of infarcering in de hersenen t/m het proximale ruggenmerg (centrale pijn) en (therapieresistente) aangezichtspijn. De neurofysiologische basis van de pijnreductie is echter nog onduidelijk en goede predictieve factoren ontbreken vooralsnog. Wel is het van groot belang gebleken om de stimulerende, negatieve, electrode zo dicht mogelijk aan te brengen boven dàt deel van de motorisch schors wat somatotopisch overeenkomt met het pijnlijke gebied. Er zijn een aantal technieken om deze plaats van te voren te bepalen zoals: functionele MRI (fMRI), Transcraniele Magneetstimulatie (TCMS) en neuronavigatie. Opvallend genoeg neemt de patiënt geen directe sensaties van stimulatie waar; de effecten op de pijn treden pas na enige tijd (minuten) op en hebben een uitdovend effect na enige uren tot soms dagen. De effectiviteit verschilt per indicatie en gerapporteerde serie maar is rond de 50% pijnreductie hetgeen in deze moeilijk behandelbare groep aanzienlijk te noemen is. Er zijn slechts beperkte studies beschreven met een langduriger follow-up. Tevens is de exacte plaats in het therapeutisch spectrum nog niet goed omschreven (4)

Ofschoon er nog veel onbekend is over het pathofysiologisch mechanisme zijn er een aantal hypothesen (4-5) op grond waarvan pijnreductie zou zijn te verklaren:

a. suppressie van thalamische neuronale activiteit
b. herstel van inhiberende baansystemen
c. toename CBF in thalamus, gyrus cinguli, frontale cortex en hersenstam
d. verandering in pijnperceptie op cognitief en affectief niveau.

Op grond van het voorgaande en de ervaring van andere centra, kunnen we de volgende indicaties voor MCS onderscheiden:

**Indicaties:**

1. centrale, neuropathische pijn [1] : unilaterale pijn t.g.v. een laesie in het CZS (thalamus) of verlengde merg (proximale myelum): pijn bij voorkeur bovenste lichaamshelft.
2. aangezichtspijn

**Het doel van ons onderzoek is tweeledig.**

1. bepalen van de haalbaarheid en klinische effectiviteit op korte, middellange en lange termijn van MCS bij centrale pijnsyndromen zoals hierboven beschreven. Een minimaal invasieve techniek verdient daarbij de voorkeur.
2. bepalen van betrouwbare (non-invasieve) predictieve factoren t.a.v. het succes van deze therapie.

Een belangrijke vraag betreft echter de werkingsmechanismen van MCS: een bijkomend resultaat van dit onderzoek kan dan ook zijn:

3. Het bestuderen van de neurofysiologische veranderingen vóór, tijdens en na stimulatie als ("objectieve") maat voor de effectiviteit van de behandeling en een mogelijke verklaring voor het pijnreducerende effect.

**Studieopzet:(**zie ook flowchart**)**

Het onderzoek is een open, observationele studie naar de effectiviteit van MCS op centrale pijn. Dit wordt o.a. bepaald door een verandering in het gebruik van analgetica en mogelijke verbetering in de QOL op korte en middellange termijn.
In een *eerste pilot fase* wordt de uitvoerbaarheid van de behandeling en het effect op de pijn bij een beperkte aantal patiënten bestudeerd. Deze pilotfase vindt in nauwe samenwerking met de afdeling Neurochirurgie van het AZG (Prof.dr.M.Staal, neurochirurg) plaats.
Na afsluiting van deze pilotfase waar de nadruk op praktische uitvoerbaarheid, veiligheid en effectiviteit ligt volgt het *vervolgonderzoek.* Patiënten worden verwezen naar de pijnpolikliniek en daar onderzocht/ geïnformeerd. Vervolgens wordt een uitgebreid consult op de polikliniek neurologie verricht.
Het voorbereidend onderzoek bestaat uit een tweetal delen. Een *poliklinisch deel* waar naast informeren van de patiënten een initiële screening op geschiktheid plaatsvindt en een neurologisch en psychologisch onderzoek plaatsvinden. Hierna zal in overleg tussen anesthesioloog, neuroloog, neurochirurg en psycholoog worden besproken of er tot voortgang van het verdere onderzoek en behandeling kan worden overgegaan.
Vervolgens een *klinisch deel* waar aanvullende diagnostiek in combinatie met een uiteindelijke operatieve ingreep kan gaan plaatsvinden.

**Patiënten selectie:**

Gezien de complexiteit van de behandeling wordt na selectie volgens onderstaande criteria een groep patiënten in de studie toegelaten in Nijmegen en Groningen.

[1] De term "centrale pijn" verwijst meestal naar pijnklachten veroorzaakt door een aantoonbare laesie in de hersenen dan wel het (proximale) myelum. De incidentie van pijn t.g.v. de eerste oorzaak is rond de 8%; myelum afwijkingen resulteren frequenter in pijnklachten 75%. De grootte van de laesie zegt niets over de intensiteit van de pijnklachten. Meestal is er een pijnvrij interval tussen diagnose en pijnklachten

Geschat wordt dat dit uiteindelijk een 10 tal patiënten per jaar in het UMC St Radboud zullen zijn.

**Inclusie criteria:**

1. therapie resistente, centrale pijn: onvoldoende effect van de gebruikelijke methodes van (pijn) behandeling (blokkades/ geen indicatie tot operatieve ingreep) naast adequaat gebruikte medicamenteuze therapie en fysiotherapie (TENS): *zie verder.*
2. aantoonbaar somatisch substraat (recente evaluatie < 3 jaar, CT scan of MRI); **Cave:** ontwikkeling syrinx.
3. ernstige neuropathische pijnklachten van centrale origine met een gemiddelde, gewogen VAS score (volgens Jensen) van 5 of hoger.
4. bereidheid tot medewerking protocol en follow-up inclusief psychologische evaluatie

**Exclusie criteria**

a. therapeutische antistolling
b. ernstige cognitieve dysfunctie en/of psychiatrische problematiek welke evaluatie hinderen.
c. (volledige) parese in pijnlijke gebied
d. nociceptieve pijn
e. epilepsie (ongecontroleerd)
f. levensverwachting < 3 jaar
g. aanwezigheid van pacemaker of ander neuromodulatie systeem
h. ernstige cardio-pulmonale co-morbiditeit/ contra-indicatie anesthesie
i. aanwezigheid van eerdere cerebrale implantaten die adequate beeldvorming belemmeren.
j. ernstige cerebrale atrofie
k. alcohol abusus (*zie verder bij evaluatie: intake poliklinisch*)

## I: Intake fase: Poliklinisch

### A: Screening/ inventarisatie vóór behandeling:

**Informatie patiënt**

- ✓ Duidelijk w.b. verwachtingspatroon
- ✓ Geef moeilijkheidsgraad therapie aan
- ✓ Voorkom teleurstelling door goede (*schriftelijke*) informatie.

**Informed Consent;** gezien het experimentele karakter van de behandeling dient naast schriftelijke informatie per patiënt informed consent verkregen te worden.

### B: Inventarisatie Pijnklachten:

Pijnklachten zijn neuropathisch en niet-nociceptief van origine: de volgende kenmerken worden bepaald:

- Pijnkarakteristieken zoals omschreven door patiënt (Pijnklachten zijn neuropathisch van origine: subjectief zie ook MPQ, bijlage):
- Pijnkarakteristiek volgens neuromodulatie screening (bijlage)
- Pijnintensiteit vlgs VAS score.

**C: Inventarisatie eerdere behandelingen**
Alvorens verdere selectie plaats kan vinden wordt gecontroleerd of navolgende therapie heeft plaatsgevonden

- **Medicatie gebruik**:
  Paracetamol, NSAID's,
  tramadol *of* buprenorphine,
  morfine *of* methadone *of* hydromorfon *of* oxycontin *of* durogesic.
  anti-depressiva,
  carbamazepine, gabapentin, (evt. lamictal/ difantoine)
  baclofen of sirdalud
  overige medicamenten: rivotril, clonidine, ketamine………………
  alcohol/drugs
- **Overige behandelingen :**
  TENS, Fysiotherapie,
  ontspanningsoefeningen/ yoga/ acupunctuur
  Operatieve ingrepen, neurochirurgische interventies,
  Neuromodulatie:
  **Andere: geef aan welke**
- **Gebruik van alcohol/ drugs/ marihuana**

**D: Algemeen lichamelijk onderzoek en neurologisch onderzoek.**
**E: Aanvullend psychologisch onderzoek en QOL onderzoek.** Hierbij hoort ook een gesprek van de partner met de psycholoog.

| | |
|---|---|
| MPQ – DLV: | Dit is de Nederlandse versie van de Mc Gill Pain Questionnaire. Verschillende aspecten van de pijn worden geïnventariseerd. |
| SCL - 90: | Vragenlijst die de lichamelijke en psychische klachten van de patiënt inventariseert. |
| Medicatiegebruik: | Inventarisatie van het medicijngebruik |
| Gewogen VAS: | Volgens Jensen. Op drie momenten op de dag, gedurende vier dagen wordt de patiënt verzocht om op een lijntje van 10 cm de pijnintensiteit aan te geven. |
| EQ - 5D: | Euro Qol. Deze lijst meet de kwaliteit van leven |
| SIP 68: | Algemene gezondheid vragenlijst |
| Pijndagboek: | In de proefbehandeling is dit dagboek gestructureerd. In de controlefasen is dit een open dagboek. |

## II: Intake: Klinische fase: op afdeling neurologie/ neurochirurgie

(zie ook bijlage flow chart bij onderzoek)

1. Transcraniele Magneetstimulatie (TMS).
2. fMRI bepaling motorisch herenschors m.b.v. MRI
3. Neuronavigatie: pre-operatieve bepaling van anatomische kenmerken op de MRI ter bepaling electrode plaatsing.
4. p.m: SSEP: toedienen van verschillende stimuli aan de extremiteiten ter beoordeling geleidingstijd zenuwstelsel/
5. p.m: RIII reflexmeting
6. p.m : QST: quantitative sensory testing

Voordat tot proefstimulatie wordt besloten wordt in consensus door de anesthesioloog, neuroloog en neurochirurg besloten tot verdere behandeling.

Op dit moment vindt overleg met de patiënt en partner/ familie plaats indien tot een operatieve ingreep wordt besloten. Indien punten 1 t/m 3 van de pre-operatieve OK screening met succes doorlopen zijn wordt overgegaan op OK-planning. Twee dagen vóór de implantatie van de epidurale elektrode kan de definitieve datum van de ingreep worden bepaald. De risico's/ belasting van de patiënt zijn vergelijkbaar met een kleine trepanatie. Het betreft een operatieve ingreep met een acceptabel risico gezien de invaliderende pijnklachten. Complicaties die zich kunnen voordoen zijn (na-) bloeding in de wond, in de epidurale ruimte binnen de schedel en infecties. Deze kunnen zowel oppervlakkig (wondinfectie) maar ook in de diepte optreden. Dit laatste is het meest risicovol gezien de kans op meningitis. Uit een grotere serie patiënten (Mertens et al: 7) zijn de risico's: infectie 2.2 %, nabloeding: 2 %, epilepsie tijdens screening 6%, follow-up: 0.7%

## III: Follow-up/ Eindpunten:

Er zijn een viertal eindpunten te herkennen:

- *Primair:*

1. Pijnreductie van 50% of meer.

- *Secundair*

2. Veranderingen in medicatiegebruik
3. Psychologische testresultaten
4. Verandering in neurofysiologische testen

**Periode van proefstimulatie.**
Gedurende de proefstimulatie periode van maximaal twee weken wordt een pijnscorelijst lijst bijgehouden. Tijdens deze twee weken wordt gestimuleerd waarbij de ideale frequentie (rond 40Hz), puls breedte (rond 82,5microsec) en amplitude (rond 2V) worden gezocht. Vanwege het onvermogen de stimulatie zelf te voelen leent deze methode zich voor een dubbelblinde evaluatie. Omdat het een potentieel risicovolle behandeling betreffende complicaties is, wordt de patiënt klinisch beoordeeld gedurende deze gehele periode. Indien tijdens stimulatie de gemiddelde VAS 50% of meer daalt en er zich geen complicaties voordoen en in consensus tot de conclusie wordt besloten dat het effect goed is, is een internalisatie van de electrode en verbinding met een pulsgenerator geïndiceerd.

**IV: Psychologische screening/ QOL; follow-up na implantatie: zie tabel 1.**
Aangezien het om een observationeel onderzoek gaat is een langdurige follow-up van belang. De follow-up is drie-vijf jaar. Deze periode wordt onderverdeeld in een aantal vaste meetpunten (zie tabel hieronder). Patiënten worden na één maand, drie, zes en negen maanden en vervolgens na een jaar gecontroleerd. Tevens is er een controle-moment na twee en drie jaar nadien.
Een goed effect is opgetreden indien de pijnscore > 50% is afgenomen t.o.v preoperatief. Afname van analgetica gebruik en verbetering van QOL zijn secundaire uitkomstmaten.
Daarnaast vindt gedurende een periode van drie jaar controle plaats van de patiënt en de effectiviteit van de behandeling, functioneren van het implantaat en registratie van technische en medisch complicaties.

**Literatuur :**

1.Tsubekowa T., Katayama Y. et al: Chronic motor cortex stimulation for the treatment of central pain. Acta Neurochir. Suppl (Wien) 1991a ; 52 :137-139.
2.Linderoth B. and Meyerson B. CNS Stimulation :in Neuropathic pain: pathophysiology and treatment; progress in pain research and management, vol 21; pp223-249.IASP Press Seattle 2001
3. Nguyen J.P, LefaucheurJ.P, Decq P. et al : Chronic motor cortex stimulation in the treatment of central and neuropathic pain. Correlations between clinical, electrophysiological and anatomical data: Pain 1999; 82: 245-251.
4. Brown J. and Barbaro N. Motor cortex stimulation for central and neuropathic pain: current status. Pain 104 (2003) 431-435.
5. Carroll D., Joint C., Maartens et al: Motor cortex stimulation for chronic neuropathic pain: a preliminary study of 10 cases. Pain 2000, 84: 431-437.
6. Garcia-Larrea L., Peyron R., Mertens P. et al. Electrical stimulation of motor cortex for pain control : a combined PET scan and electrophysiological study . Pain 1999; 83: 259-273.
7. Canavero S, Bonicalzi V. The neurochemistry of central pain: evidence from clinical studie, hypothesis and therapeutic implications. Pain 1998; 74: 109-114.
8. Mark P. Jensen, Judith A. Turner, Joan M. Romano and Lloyd D. Fisher Comparative reliability and validity of chronic pain intensity measures. Pain, 1999; 83 (2)157-162 .
9. Mertens P. Motor cortex stimulation in the treatment of central neuropathic pain: voordracht: Central Pain 24-5-2003, Amsterdam.
7. Central pain states; R.Tasker in Bonica's management of pain 3rd edition J.Loeser (ed.) 433-457.

**Bijlage 1.**
**Screening formulieren.**

MPQ - DLV: Dit is de Nederlandse versie van de Mc Gill Pain Questionnaire. Verschillende aspecten van de pijn worden geïnventariseerd.

SCL - 90: Vragenlijst die de lichamelijke en psychische klachten van de patiënt inventariseert.

Medicatiegebruik: Inventarisatie van het medicijngebruik

Gewogen VAS: Volgens Jensen. Op drie momenten op de dag, gedurende vier dagen wordt de patiënt verzocht om op een lijntje van 10 cm de pijnintensiteit aan te geven.

EQ - 5D: Euro Qol. Deze lijst meet de kwaliteit van leven

SIP 68: Algemene gezondheid vragenlijst

Pijndagboek: In de proefbehandeling is dit dagboek gestructureerd. In de controlefasen is dit een open dagboek.

Tabel 1

| | Intake fase | Proefbehan-deling fase | Implantatie & post-operatieve | Controle na 1 maand | Controle na 3 maanden | Controle na 6 maanden | Controle na 9 maanden | Controle na 12 maanden | Controle na 18 maanden |
|---|---|---|---|---|---|---|---|---|---|
| MPQ-DLV | X | | | | X | | X | | X |
| SCL-90 | X | | | | | | | | |
| Medicatiegebruik | X | | | X | X | X | X | X | X |
| Gewogen VAS | X | X | | X | X | X | X | X | X |
| Kwaliteit van leven | X | | | | | X | | X | X |
| Algemene gezondheid | X | | | | | X | | X | X |
| Pijndagboek | | (X) | | X | X | X | X | X | X |

### Bijlage 2
## Sensory Testing for Motor Cortex Stimulation Studies

Quantitative Sensory Testing

**A. ELECTRICAL STIMULATION**

***Stimulus characteristics***

- transcutaneous electric stimulation
- sensation, pain detection and pain tolerance thresholds
- single and repeated stimuli
- in the area of maximum pain and in an area far from the pain, followed by VAS rating of a series of suprathreshold stimuli in both these areas.

***Sites of testing***

- Within area of pain for which MCS was instituted and contralateral homologous area
- Within area far from pain area for which MCS was instituted (T4 dermatome, mid-axillary line, ipsi- and contralateral to side of MCS stimulation)
- Within area far from pain area for which MCS was instituted (L1 dermatome, iliac crest, ipsi- and contralateral to side of MCS stimulation)

B. MECHANICAL STIMULATION

***Stimulus characteristics***

- Somedic algometer
- pain detection thresholds

**Sites of testing**

- Within area of pain for which MCS was instituted and contralateral homologous area
- Within area far from pain area for which MCS was instituted (T4 dermatome, mid-axillary line, ipsi- and contralateral to side of MCS stimulation)
- Within area far from pain area for which MCS was instituted (L1 dermatome, iliac crest, ipsi- and contralateral to side of MCS stimulation)

C. MICROFILAMENT STIMULATION

***Stimulus characteristics***

- Von Frey hair threshold determination and mapping

**Sites of testing**

- allodynic/hyperalgesic area for which MCS instituted

R-III reflex

Electrical stimuli will be delivered percutaneously to the sural nerve using two self-adhering surface electrodes placed behind the lateral malleolus (Willer 1977; Hugon 1973; Skljarevski & Ramadan 2002). The stimulus will be a constant current rectangular pulse train consisting of five pulses, each of 1 ms and delivered at 200 Hz by a computer controlled constant current stimulator. The stimulus intensity will be 1.2 times the reflex threshold intensity, defined as the lowest stimulus intensity evoking reflexes in 3 successive tests. The reflex threshold is defined as EMG activity above 20 μV for a period of at least 5 ms in the 70-200 ms post-stimulus interval.

The electromyogram (EMG) will be recorded from the ipsilateral biceps femoris. Prior to the electrode attachment, the skin will be lightly abraded and cleaned with isopropyl alcohol. The EMG signals will be filtered (20-500 Hz; 4th order), amplified, and sampled into a computer (2 kHz), monitored on a computer storage oscilloscope, and stored on **disk for later analysis.** EMG sweeps of 1 s are stored from 200 ms prior to the electrical stimulation. The EMG sweeps will be rectified and averaged (8 stimulations), with an inter-stimulus interval (ISI) of at least 15 s. The Root-Mean-Square (RMS) amplitude will be measured in the interval 70-200 ms after the electrical stimulation as an indicator of the size of the nociceptive withdrawal reflex (RIII-reflex; Hugon 1973). A summation threshold will also be determined by the evoked EMG response by delivering five electrical stimuli with an ISI of 500ms. The EMG-summation threshold is defined as the lowest stimulus intensity where the mean size of the two last reflexes is 20% higher than the mean reflex size of the first three reflexes. Further, the size of the last reflex has to be above 20μV for a period of at least 5 ms in the 70-200 ms post-stimulus interval.

<u>Somatosensory Evoked Potentials</u>
Electrical stimuli will be delivered percutaneously to the posterior tibial nerve using two self-adhering surface electrodes at the lowest twitch level on the abductor hallucis brevis muscle. The nerve is stimulated at the ankle, with a stimulus electrode (interelectrode distance 2.5 mm) The posterior tibial nerve is stimulated with monophasic square pulses, 0.2 milliseconds in duration at a rate of 4 Hz, 1000/averages, bandwidth 20–1 kHz.

Electrode montage:

- fossa poplitea- biceps femoris tendon
- Thoracic 12-iliac crest
- C3'-Fz
- Cz-Fz

**Bijlage 3:**
**Transcraniele magneetstimulatie**
Door middel van een uitwendige, niet-pijnlijke magneetstimulatie wordt de (motorische) hersenschors gelocaliseerd waar de epidurale electrode gepositioneerd moet worden. Dit onderzoek vindt plaats enerzijds ter voorspelling van de lokalisatie van de electrode, anderzijds ter bepaling van een gunstig effect bij implantatie.

**Bijlage 4.**
**Chirurgische techniek**:
Risico's/ belasting van de patiënt: deze zijn vergelijkbaar met een kleine trepanatie. Het betreft een operatieve ingreep met een acceptabel risico gezien de invaliderende pijnklachten
<u>Opzet:</u>
-Gebruik van Image Guidance System voor localisering van de motore hersenschors.
-Exacte pre-operatieve lokalisering van (motore) gebied dat gestimuleerd moet worden d.m.v.
    -fMRI
    -TransCraniële Magneet Stimulatie (TCMS)
-Doel hiervan is het mogelijk maken van kleine, doch sufficiënte chirurgische toegang tot het doelgebied.

-Voordeel van deze methode is het ontbreken van noodzaak tot intra-operatieve neurofysiologische lokalisering. Edoch intraoperatieve stimulatie is nog wel noodzakelijk.

1) Bij gebruik TMS als lokalisering methode wordt met TMS het gewenste cortex gebied bepaald, waarna het punctum maximum met een navigatie-fiducial wordt gemarkeerd. Vervolgens gaat patiënt met de fiducial(s) naar de MRI voor het maken van een plannings MRI, welke de volgende dag bij de operatie als geleider van de electrode wordt gebruikt.
2) Bij gebruik van fMRI als lokaliserings methode krijgt patiënt een fMRI direct gevolgd door een plannings MRI, waarna beide modaliteiten “ gemerged “ worden om zo functie in anatomie te integreren. De operatie vindt de volgende dag op geleide van de navigatie MRI plaats.

Operatieve ingreep:

1) onder algehele anesthesie; positionering patiënt in Mayfield schedel klem
2) na opbouwen 3D model bepalen van een kleine craniotomie over centrale sulcus, met geplande lokatie in centrum.
3) Craniotomie als gebruikelijk
4) Plaatsen epidurale electrode op geplande plaats.
5) Proefstimuleren met aanpassing narcose om motore effect gebied te controleren.
6) Evt aanpassing van plaats elektrode.
7) Fixatie electrode met 2 hechtingen aan dura.
8) Terugplaatsen botlap, met uitleiden elektrode door boorgat
9) Subcutaan naar buiten tunnelen van elektrode, rekening houdend met latere interne traject van elektrode!
10) Sluiting als gewoonlijk.

Postoperatieve periode:

Proefstimulatie gedurende twee weken, waarbij de ideale frequentie (rond 40Hz), puls breedte (rond 82,5microsec) en amplitude (rond 2V) worden gezocht. Indien > 50% pijnreductie wordt bereikt wordt van succesvolle stimulatie gesproken en wordt tot implantatie van het systeem overgegaan, met positionering van de batterij onder ipsilaterale clavicula. Bij onvoldoende reactie na dezelfde periode verwijdering van het systeem.

Noot:

-controle radiologie ter controle juiste positie elektrode met X-schedel/ MRI.
